# Marine Bioactive Substances in Precision Nutrient Delivery to the Gut and Advances in Microbiome Regulation: A Narrative Review

**DOI:** 10.3390/foods15030545

**Published:** 2026-02-04

**Authors:** Xue Zhao, Shan Huang, Ya Wei, Di Wang, Chunsheng Li, Chuang Pan, Yueqi Wang, Huan Xiang, Gang Yu, Yongqiang Zhao

**Affiliations:** 1 Key Laboratory of Aquatic Processing, Ministry of Agriculture and Rural Affairs, National Research and Development Center for Aquatic Product Processing, South China Sea Fisheries Research Institute, Chinese Academy of Fishery Sciences, Guangzhou 510300, China; zhaoxue@scsfri.ac.cn (X.Z.); 21240713121@stu.ouc.edu.cn (S.H.); weiya@scsfri.ac.cn (Y.W.); wangdi@scsfri.ac.cn (D.W.); lichunsheng@scsfri.ac.cn (C.L.); panchuang@scsfri.ac.cn (C.P.); wangyueqi@scsfri.ac.cn (Y.W.); xianghuan@scsfri.ac.cn (H.X.); 2 Key Laboratory of Efficient Utilization and Processing of Marine Fishery Resources of Hainan Province, Sanya Tropical Fisheries Research Institute, Sanya 572426, China; 3 College of Food Science and Engineering, Ocean University of China, Qingdao 266000, China

**Keywords:** marine bioactive substances, gut microbiota regulation, precision nutrition, delivery systems, bioavailability

## Abstract

Marine bioactive substances exhibit structural diversity and function-specific properties, attracting considerable interest in their potential applications in targeted nutritional delivery to the gut and microbiota regulation. These bioactive components, sourced from seaweed, marine crustaceans, and microorganisms, including polysaccharides, polyphenols, and lipids, demonstrate exceptional biocompatibility and specific recognition capabilities. They serve as an optimal carrier matrix and functional core for developing an efficient, precision-targeted intestinal nutrition delivery system. Research findings demonstrate that optimization via innovative delivery technologies, including nanoencapsulation and polymer microsphere encapsulation, enables marine bioactive substances to navigate various physiological barriers in the gastrointestinal tract effectively. This facilitates targeted, sustained release of nutritional components and enhances bioavailability. Simultaneously, these substances may relieve dysbiosis by modulating the composition of the gut microbiota and the quantity and activity of specific metabolic products, thereby reinforcing intestinal barrier integrity. This narrative review systematically examines the sources and functional attributes of marine bioactive compounds, emphasizing their application strategies in developing targeted delivery systems for the gut and their regulatory effects on gut microbiota. It concludes by delineating future research directions in this field, particularly in optimizing carrier functionalities and clarifying action mechanisms.

## 1. Introduction

As a crucial organ for digestion, absorption, and immune defense, the gut’s microbial equilibrium and precise nutrient utilization significantly impact human health [1]. With the swift progress in functional foods, the regulation of gut microbiota and the enhancement of nutrient efficiency through the precision delivery of marine bioactive substances have become pivotal research areas in food science and nutrition [2]. Conventional bioactive compounds sourced from marine environments frequently encounter issues such as low bioavailability and inadequate precision in intestinal delivery. To alleviate these challenges, novel precision nutrition delivery strategies have been outlined [3]. Marine organisms, having adapted to prolonged exposure to extreme conditions such as high pressure, low temperatures, and elevated salinity, have developed unique metabolic pathways. Consequently, the bioactive compounds they produce possess distinct chemical structures and physiological functions compared to those from other origins. Through innovative application strategies, these compounds exhibit remarkable biocompatibility, low toxicity, and enhanced intestinal specificity, thereby establishing a research foundation for the development of precision nutrition delivery systems and the regulation of the gut microbiome [4].

In the progressive advancement of functional foods, marine-derived bioactive compounds are emerging as a promising vehicle in gut nutrition regulation, capitalizing on their intrinsic natural benefits [5]. In contrast to conventional single-delivery methods, the utilization of marine bioactive substances to fulfill the nutritional enhancement needs of functional foods facilitates novel precision nutrition delivery strategies that amplify the specific interaction between these substances and the intestinal microenvironment [6]. This approach allows for the precise release and effective absorption of nutrients within the gut. Research indicates that nutrient delivery systems based on marine bioactive compounds can effectively endure the acidic conditions of the stomach and the degrading effects of digestive enzymes, thereby significantly enhancing the structural integrity and bioavailability of active ingredients upon reaching the intestines [7]. Concurrently, these bioactive substances can stimulate the proliferation of beneficial bacteria and inhibit the growth of pathogenic bacteria by modulating the abundance and diversity of the gut microbiota, thus preserving the dynamic equilibrium of the intestinal microecosystem [8]. This dual functionality of precise delivery and microecological regulation is essential for advancing functional foods from mere nutritional supplementation to individualized nutritional health regulation [9]. Marine polysaccharides, polyphenols, and lipids, among various marine bioactive substances, exhibit remarkable biocompatibility and gut-specific recognition, making them the focal point of precision nutrition delivery research in the gastrointestinal tract [10,11,12]. The molecular structure of marine polysaccharides is abundant in active functional groups, such as hydroxyl and carboxyl groups, which confer exceptional water solubility and film-forming capabilities. Through interactions and modifications, these polysaccharides can bind to specific receptors on intestinal epithelial cell surfaces, facilitating precise nutrient delivery [13]. For instance, sulfated fucoidan derived from brown algae can extend nutrient retention time in the intestine by adhering specifically to the intestinal mucosal epithelium, thereby significantly improving absorption efficiency [14]. Marine polyphenols contain phenolic hydroxyl groups in their molecular composition. These groups not only impart significant antioxidant properties but also allow for specific interactions with certain enzymes generated by gut microbiota, thereby promoting the specified release of active constituents within the intestinal tract [15]. Marine lipids such as polyunsaturated fatty acids in fish oil and astaxanthin esters can form nanoemulsions or encapsulate nutrients within liposomes. By exploiting the homology between lipids and intestinal cell membranes, they attain effective penetration while modulating the synthesis of gut microbiota metabolites, therefore contributing to the dynamic regulation of intestinal health [16].

Although marine bioactive substances have achieved a series of breakthroughs in the fields of precision nutritional delivery and microbiota regulation within the gut, this area still faces numerous scientific challenges and technical bottlenecks that require urgent resolution. For instance, the mechanisms of action for some bioactive substances remain unclear [17], while the stability of delivery systems and the difficulties associated with large-scale production pose significant obstacles [18]. Marine-derived bioactive substances demonstrate significant advantages over terrestrial equivalents in critical performance metrics. Specifically, marine polysaccharides-such as alginates, chitosan, and carrageenan-feature distinctive functional groups (sulfate, carboxyl, hydroxyl) that facilitate the formation of three-dimensional cross-linked networks, achieving encapsulation efficiencies of 80–95% for active compounds. Conversely, terrestrial polysaccharides (cellulose, starch, guar gum) exhibit limited water solubility due to their densely packed crystalline structures, typically resulting in encapsulation efficiencies below 60% [19]. Furthermore, marine-derived agarose and carrageenan enhance delivery efficacy by establishing stable three-dimensional networks, thereby improving the stability of encapsulated small-molecule components [20]. Conversely, branched starch and linear starch often engage in delivery mechanisms. Linear starch features a linear, unbranched configuration, which diminishes its solubility in water and facilitates the creation of more stable gels. In contrast, branched starch has a branched structure, enhancing its solubility in water and producing gels with lower viscosity and diminished stability [21]. Marine-derived bioactive substances provide significant benefits for microbiome modulation due to their resilient physical structure and protective barrier against extreme gastrointestinal conditions. Their influence on the microbiome likely functions mainly as prebiotics or by physically modifying the intestinal environment. For example, macroalgal polysaccharides enhance the intestinal barrier, modulate gut microbiota composition, affect metabolic processes, and regulate immune responses [22]. Conversely, the function of terrestrial-derived carriers transcends mere physical protection, highlighting active biological regulation. For example, polyphenols exhibit various bioactivities-including antioxidant, anti-inflammatory, and selective antibacterial properties (suppressing pathogens while enhancing probiotics)-that synergize with probiotics. They directly influence the intestinal microenvironment, host gene expression, and immune responses, thus facilitating more precise microbiome regulation [23]. Notwithstanding the benefits of marine-derived bioactive substances, notable limitations persist. For example, marine polysaccharides necessitate modification owing to inadequate mechanical properties and exhibit rapid degradation under physiological conditions. Furthermore, marine raw materials demonstrate considerable batch-to-batch variability, with their physicochemical properties significantly affected by species, geographic origin, and extraction season, complicating the standardization of industrial production [24]. Moreover, although bioactive compounds from marine sources typically demonstrate biocompatibility, significant contradictions arise. For example, potential allergenicity presents a risk; chitin sourced from crustacean exoskeletons may induce allergic reactions in individuals with shellfish sensitivities [25]. Concurrently, there are knowledge gaps in the existing research on marine-derived bioactive compounds. Specifically, the majority of applications of sulfated polysaccharides are restricted to in vitro studies, with scant in vivo and clinical data accessible. Consequently, additional research is imperative to ascertain their safety and efficacy within biological systems [26].

**Figure 1:**
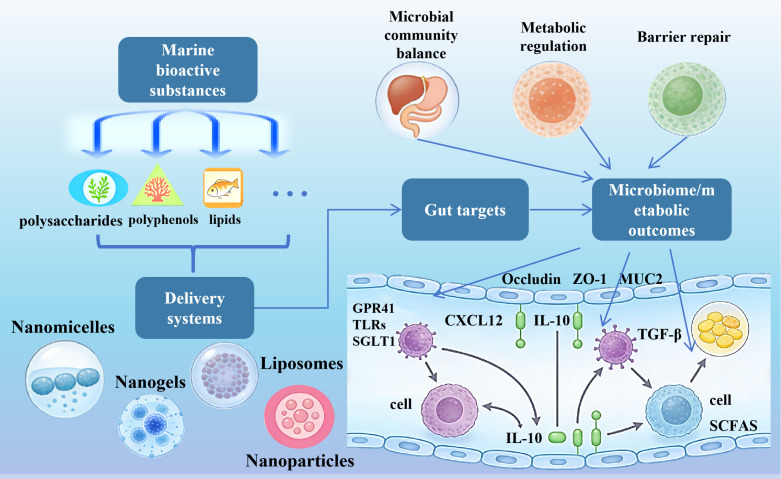
Schematic diagram illustrating the pathway by marine bioactive substances targeting the gut via delivery systems to regulate the microbiome and metabolism.

Therefore, systematically organizing the application models of marine bioactive substances in precision nutritional delivery to the gut and thoroughly elucidating their regulatory mechanisms on the gut microbiota holds significant practical value for advancing the innovative research and development of functional foods [27]. Therefore, this paper will focus on three representative bioactive compounds-marine polysaccharides, polyphenols, and lipids-to systematically review their application strategies in constructing precision nutritional delivery systems for the gut. It will also conduct an in-depth analysis of their regulatory effects on gut microbiota function [28], comprehensively summarize the key issues in current research, and outline future development directions, providing a reference for the high-value utilization of marine biological resources and the precision development of functional foods (Figure 1) [29].

## 2. Mechanism of Marine Polysaccharides

### 2.1. Structural Characteristics and Intestinal Tolerance

Marine polysaccharides are natural polymer compounds extracted from marine organisms, including alginates, carrageenans, chitin, agar, and fucoidans (Table 1). The unique environmental conditions of marine ecosystems (high salinity, high pressure, low temperatures, etc.), as well as heterogeneity in marine polysaccharides, significantly distinguish them from terrestrial polysaccharides. This structural characteristic is the core determinant of their intestinal tolerance [30,31]. The monosaccharide composition of marine polysaccharides is highly diverse. In addition to common monosaccharides, such as glucose and galactose, they contain notable monosaccharides like fucose, mannose, and galacturonic acid. For example, alginic acid consists of linear chains formed by alternating β-D-mannuronic acid and α-L-guluronic acid units linked via 1,4-glycosidic bonds. Carrageenan, which is based on galactose residues, exhibits a unique helical conformation due to modifications by sulfate groups [32,33]. The differences in glycosidic bond types further enhance structural specificity. The alternating β-(1→3) and β-(1→4) glycosidic bonds in agar reinforce its molecular rigidity, whereas the presence of multiple glycosidic bonds, such as α-(1→2) and α-(1→3), in fucoidan results in its rich branched structure [34,35]. As a result, they demonstrate remarkable intestinal tolerance and can arrive at the large intestine unaltered [36]. Furthermore, the polar functional groups on the surfaces of marine polysaccharide molecules—such as hydroxyl and carboxyl groups—can engage in hydrogen bonding with water molecules, thereby forming stable colloidal solutions. This physicochemical characteristic not only improves their stability within the intestinal environment but also provides a structural basis for subsequent interactions with gut microbiota [37].

### 2.2. Intestinal Targeted Delivery Properties and Mechanism of Action

**Figure 2:**
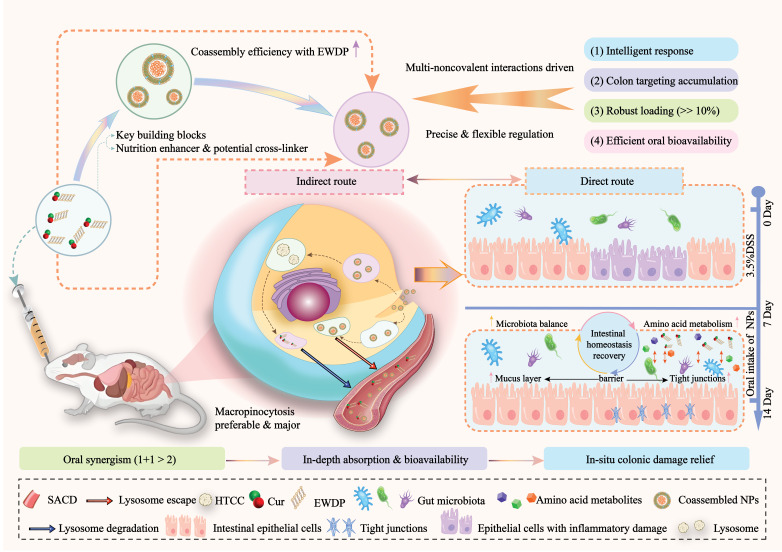
Schematic illustration of the programmable food-derived peptide co-assembly strategies for boosting orally targeted colitis therapy. Egg white-derived peptides could function as both potential cross-linkers and nutrition enhancers to regulate the co-assembly process of NPs. After oral administration, NPs could achieve the preferable oral synergism in an indirect and direct manner, including the enhanced in-depth oral bioavailability of nutraceuticals via the macropinocytosis pathway and multiple in situ interactions between NPs and the colonic microenvironment.

Marine polysaccharides, derived from oceanic sources, serve as natural macromolecular carriers that facilitate precise intestinal delivery due to their well-aligned structure and function. The structure-activity relationships and delivery system design of representative examples such as alginate, chitosan, and carrageenan have become key research focuses [38]. The acid and enzymatic barriers in the gastrointestinal tract impose stringent demands on carrier stability, while the structural heterogeneity of marine polysaccharides endows them with inherent resistance: the acid-resistant core of alginate forms an “egg carton” structure composed of glucuronic acid (G) residues. An increased proportion of G units leads to a denser gel network, and hydrophobic modification further facilitates pH-responsive delivery of fucoxanthin [39]. Nano-chitin, characterized by its nanoscale particle size and elevated specific surface area, serves dual roles as a lipid regulator and active carrier [40]. Colon-targeting nanoparticles (NPs) composed of a quaternary ammonium chitosan (HTCC) shell and succinic acid-modified γ-cyclodextrin core for precise interventions for colitis (Figure 2) [41]. For precise intestinal inflammation intervention strategies, chitosan-ferritin complex stabilized Pickering emulsion can also be used to co-encapsulate active substances [42]. The sulfate groups of carrageenan enhance system stability through electrostatic interactions, and its composite nanoparticles have successfully achieved efficient encapsulation of soy aglycones [43]. Nanoparticles composed of low-melting-point agarose enhance the functionality of DHA in functional foods through the matrix’s sustained-release properties [44]. Ulvan from stonewort can improve the gelatinization properties of rice noodles, enhancing their quality by increasing viscosity and gel density. They also regulate emulsion stability in food systems and extend the shelf life of oil-containing foods [45]. These structure-activity relationship-based designs fully tap the potential of marine polysaccharides, offering new pathways for the efficient application of marine-derived carriers [46].

**Table 1:** The main categories of marine polysaccharides.

Polysaccharide Name	Applications	Ref.
Alginate	Hydrophobically modified alginate enables pH-responsive targeted delivery of fucoxanthin	[47]
Extracellular Polysaccharide (EPS)	EPS from microalga Parachlorella sp. BX1.5 (derived from edible strains) can be used as an anti-hypertensive food ingredient	[48]
Chitin	Nano-chitin can serve as a food lipid regulator and active substance carrier	[49]
Carrageenan	Carrageenan composite nanoparticles loaded with daidzein for food active substance delivery	[43]
Chitosan	Chitosan composite films for preserving semi-dried golden pomfret and extending shelf life	[50]
Chitosan Oligosaccharide (COS)	COS-ferritin complexes stabilizing Pickering emulsions for co-encapsulation of active substances	[51]
Chondroitin Sulfate	Chondroitin sulfate nanoparticles encapsulating pigments for fish preservation and freshness indication	[52]
Agar	Low-melting agarose composite nanoparticles encapsulating DHA to improve functional food applicability	[53]
Ulvan	Ulvan improves the gelatinization properties of rice flour, enhancing viscosity and gel compactness to optimize the quality of rice flour products; it regulates the emulsification stability of food systems and extends the shelf life of oil-containing foods	[10]
Fucoidan	Fucoidan/chitosan nanoliposomes co-encapsulating food active phenolics	[54]

### 2.3. Dual Regulatory Pathways of the Gut Microbiome

Marine polysaccharides, as bioactive macromolecules exclusive to marine organisms, exhibit structural characteristics that fundamentally regulate intestinal microbiota [55]. This structural advantage provides remarkable stability and biocompatibility within the intestinal ecosystem [56].

Marine polysaccharides exhibit prebiotic effects by demonstrating non-digestibility, fermentability, and pathogen-inhibiting potential, thereby enhancing the activity of gut microbiota [57]. On the mechanism of direct microbiota modulation, fucoidan possesses an α-(1→3)-L-fucose backbone with β-(1→4) glycosidic bonds linking branched galactose residues. When its branching degree reaches ≥30%, it is efficiently recognized by *Bacteroides* species. The direct modulation of marine polysaccharides on the intestinal microbiota is contingent upon specific structural interactions with microbial communities. Their saccharide chain configurations act as selective carbon sources for gut microbiota, promoting the proliferation of beneficial bacteria through structural-substrate compatibility [58]. The heteropolysaccharide composition of microalgal extracellular polysaccharides (EPS) encompasses specific aldonic acid residues that are distinctly recognized by lactobacillus glycosidases, facilitating the production of short-chain fatty acids to regulate the intestinal microenvironment [59]. On the barrier effect mechanism, the indirect regulatory pathway optimizes structure-mediated intestinal barrier function. The sulfated glucuronic acid repeating units of Ulvan polysaccharides augment the gelatinization density of rice flour. In the preservation of oil-containing foods, they strengthen the physical barrier by forming hydrogen bonds with serine residues in intestinal mucins [60].

On the mechanism of delivery-mediated effects, the chitosan hydrogel delivery system not only achieved a sustained release of probiotics exceeding 10^7^ colony-forming units (CFU)/g (Table S1) in a simulated gastrointestinal environment, but also ensured a survival rate of at least 71.3% and 55.1% of probiotics after prolonged storage for 28 days at −20 °C and 4 °C, respectively [61]. Furthermore, the chitosan-formed nanoliposomes enhance astaxanthin retention in the intestine due to their structural complementarity [62]. Phenolic acid grafting transformed agarose from a hydrophilic gel to an amphiphilic latex, which significantly improved the encapsulation efficiency, chemical stability, and bioavailability of lutein [63]. Nanoparticles consisting of 50–100 kDa components, upon encapsulating DHA, can create a protective layer on the intestinal mucosa, thereby improving the bioavailability of functional components [64]. Chondroitin sulfate, when delivered via nanocarrier technology, reduces the expression of inflammatory markers in vitro [65]. These regulatory pathways, which collectively form a network, center on marine polysaccharides and precisely modulate the gut microbiome, providing theoretical support for functional food development [66].

## 3. Mechanisms of Interaction Between Marine Polyphenols and Lipids

### 3.1. Characteristics and Activity of Marine Polyphenols and Lipids

#### 3.1.1. Advantages of Functional Components and Sources of Marine Polyphenols

Marine polyphenols represent a category of secondary metabolites extensively found in marine algae. The number and arrangement of phenolic hydroxyl groups in their molecular structures, coupled with the presence of conjugated systems, endow them with exceptional antioxidant and surface-active properties [67]. Marine polyphenolic compounds with high application value in the field of functional foods primarily include brown algae polyphenols and fucoxanthin, which possess polyphenolic characteristics. The structural differences between these two compounds determine their respective distribution ranges and application directions [68].

Brown algae polyphenols are polyphenolic polymers exclusive to brown algae, characterized by resorcinol as their fundamental structural unit. They generate linear or branching macromolecules through covalent bonding at different degrees of polymerization [69]. The elevated concentration of phenolic hydroxyl groups on their molecular surfaces serves as their principal active sites [70]. Seaweeds have a specific class of polyphenols, the phlorotannins, which are polymers of phloroglucinol (1,3,5-trihydroxybenzene). They exhibit high biological activities, including antioxidant, antibacterial, antifungal, anti-inflammatory, and neuroprotective activities. Although marine polyphenols have shown good bioavailability, it should be noted that polyphenols have low bioavailability, especially after oral administration, and in vitro activity may differ. This accessibility can be further enhanced through encapsulation, carrier development, and delivery systems [71]. Phlorotannins form the primary component of a natural composite wall material for the microencapsulation of functional lipids. Research has demonstrated that phlorotannins can create a ternary composite wall material in conjunction with pea protein isolate and chitosan for the microencapsulation of tomato seed oil. This system leverages the cross-linking properties and interfacial activity of brown algae polyphenols to substantially enhance microencapsulation efficiency and storage stability. In accelerated oxidation experiments, the increase in peroxide value of the encapsulated tomato seed oil was merely one-fifth that of the unencapsulated control group, thereby providing essential technical support for the stable application of functional oils in the food industry [72].

Fucoxanthin is classed taxonomically as a carotenoid. The presence of polyene conjugated double bonds, epoxy groups, and hydroxyl functional groups in its molecular structure imparts antioxidant action similar to that of polyphenolic substances. Thus, it is characterized as a marine bioactive compound demonstrating polyphenol-like properties [73]. Fucoxanthin demonstrates particular localization within photosynthetic tissues, predominantly found in the photosynthetic cells of brown algal thalli and on the thylakoid membranes of marine microalgae, including *Haematococcus pluvialis* and *Chlorella vulgaris*. *Haematococcus pluvialis* is an exceptionally proficient generator of fucoxanthin. In artificial photobioreactor cultivation conditions, the intracellular fucoxanthin level can attain 18.7 mg/g dry weight [74]. Moreover, fucoxanthin sourced from marine microalgae demonstrates a more stable conjugated double bond structure, with antioxidant activity 2.5 to 3.0 times greater than that of terrestrial carotenoids [75]. An investigation developed a N-acetylgalactosamine-modified probiotic vesicle delivery system to overcome the delivery penetration barrier in alcoholic liver disease and to enhance fucoxanthin-mediated cholesterol-lowering therapy for the improvement of alcohol-induced lipid metabolic disorders. This targeted modification increased the encapsulation efficiency of fucoxanthin in the *Lactobacillus plantarum* Lp90-derived nanovesicle delivery system to 84.81% and significantly improved the water solubility of fucoxanthin. Furthermore, it exhibited substantial resistance to degradation by gastrointestinal fluids. In the HepG2 cell model, the method achieved a 2.52-fold increase in uptake by specifically targeting the asialoglycoprotein receptor [76]. A ternary complex consisting of whey protein isolate, sodium alginate, and seaweed-derived polyphenols was formulated as an emulsifier to stabilize water-in-oil emulsions. The small average droplet size (428.8 ± 5.4 nm) and significantly negative ζ-potential (−47.1 ± 1.1 mV) demonstrated superior colloidal stability. The ternary emulsion exhibited enhanced inhibition of free fatty acid (FFA) release compared to binary and single-component systems during simulated gastrointestinal digestion. The liberation of free fatty acids during the intestinal phase diminished by approximately 15%, whereas no notable release occurred in the gastric phase. The antioxidant capacity of the ternary system in the intestinal phase was double that of the binary emulsion and over threefold that of the emulsion solely stabilized by whey protein isolate. This system exhibits substantial potential for utilization in functional foods and nutraceuticals for efficient delivery [77].

#### 3.1.2. Advantages of Functional Components and Sources of Marine Lipids

Marine lipids denote lipid-based bioactive compounds obtained from marine organisms, primarily composed of highly unsaturated fatty acids and carotenoid-type lipids. These lipids present unique benefits in molecular structure, sustainability of source, and nutritional compatibility, establishing them as essential ingredients for food fortification and functional delivery systems [78]. Currently, the marine lipid functional components with established applications in the food industry are DHA (docosahexaenoic acid) [79] and astaxanthin [80]. Their structural characteristics and marine-sourced advantages collectively drive their industrialized application in the functional food sector [81].

DHA is a typical omega-3 long-chain polyunsaturated fatty acid. Its molecular structure contains six cis-unsaturated double bonds arranged in a non-conjugated pattern. This structure endows it with both excellent nutritional activity and molecular compatibility. Additionally, the specific arrangement of these double bonds optimizes their metabolic absorption within the body [82]. From a sourcing perspective, marine ecosystems function as natural reservoirs for DHA, which can be classified into two principal categories: marine animal-derived and microalgae-derived [83]. Marine animal-derived DHA is predominantly extracted from the muscle and visceral tissues of deep-sea fish, such as tuna [84] and cod [85]. Conversely, marine microalgae-derived DHA is produced using *Schizochytrium* sp. and *Crypthecodinium cohnii* as primary production strains [86]. Microalgal DHA presents considerable sourcing advantages, as its production process is independent of marine fishery resources and allows for specific synthesis via fermentation. Furthermore, DHA derived from microalgae is devoid of the anti-nutritional factors typically associated with terrestrial lipids, thereby substantially improving its biosafety and compatibility with food [87]. Inspired by the fat globule membrane structure found in breast milk and infant formula, liposomes of varying sizes and compositions were developed for the delivery of lactoferrin and DHA. In vitro simulations of infant semi-dynamic digestive behavior and liposome absorption in intestinal organoids demonstrated minimal structural alterations in liposomes, while degradation was observed within the intestine. The degradation rates of liposomes were predominantly affected by particle size, with composite phospholipids facilitating DHA hydrolysis. The release rate of DHA from small-volume liposomes surpassed that of free DHA. This research offers new perspectives on the membrane structure-function relationship of liposomes and may aid in the creation of innovative functional foods for kids [88]. This methodology establishes a technical framework for the development of foods with synergistic fortification of various nutrients [89].

Astaxanthin is a red carotenoid lipid characterized by a molecular structure with polar ends formed by ketone and hydroxyl groups, and a hydrophobic core consisting of a conjugated double bond chain. This distinctive amphiphilic structure provides it with antioxidant properties and targeted delivery capabilities [90]. The primary source of marine astaxanthin is the dormant spores of *Haematococcus pluvialis*, which predominantly exhibit the 3S, 3’S configuration, demonstrating antioxidant activity 1.8 to 2.2 times superior to that of chemically synthesized alternatives, while also mitigating food safety risks associated with configuration impurities. Moreover, natural astaxanthin is found in Antarctic krill (*Euphausia superba*) [91]. The benefits of marine-derived astaxanthin are evident in its sustainable mass production. Cultivated in closed photobioreactors, *Haematococcus pluvialis* attains an astaxanthin production efficiency of up to 45 7 mg/g dry weight. This cultivation process facilitates carbon recycling, conforming to the ecological production standards of the food industry [92]. Astaxanthin, due to its amphiphilic structure, can be encapsulated within oral liver-targeted nanoparticles. These nanoparticles utilize the synergistic interaction between astaxanthin’s polar terminal group and the carrier’s targeting ligand to significantly improve their enrichment efficiency in specific digestive segments. In simulated digestive systems, the targeted retention rate of these nanoparticles achieves 76.2%, effectively ensuring the stability and bioactivity of astaxanthin in functional foods [93].

Furthermore, marine lipids provide a unique advantage as their artificial cultivation systems for marine microalgae allow for precise customization of functional lipids. The molecular structure of these products demonstrates natural compatibility with human lipid metabolic pathways, and the distribution of double bonds in unsaturated fatty acids enhances recognition by digestive enzymes [94]. This attribute broadens their applicability in nutritional foods for specific populations. In conclusion, the structural characteristics of marine polyphenols and lipids are central to their bioactivity expression [95,96]. Meanwhile, resource sustainability and component specificity derived from marine origins position them as core carriers for developing high-value functional foods. The precise development and application of these two components will provide critical support for the high-value conversion of marine biological resources into food products [97,98]. Marine lipids and marine polyphenols are crucial functional elements in precision nutrient delivery (Table 2). Marine lipids, known for their excellent biocompatibility and high bioavailability, are often used as nanocarrier matrices. Marine polyphenols, which have antioxidant properties and the ability to target specific areas, can modify delivery surfaces to improve targeting efficiency. They also work with nutrients to reduce the biotoxicity of delivery systems. Therefore, the combined delivery systems created with these two components have significant potential in the functional food industry, offering highly effective solutions for precision nutritional interventions.

### 3.2. The Functions of Marine Polyphenols in Delivery Systems

**Figure 3:**
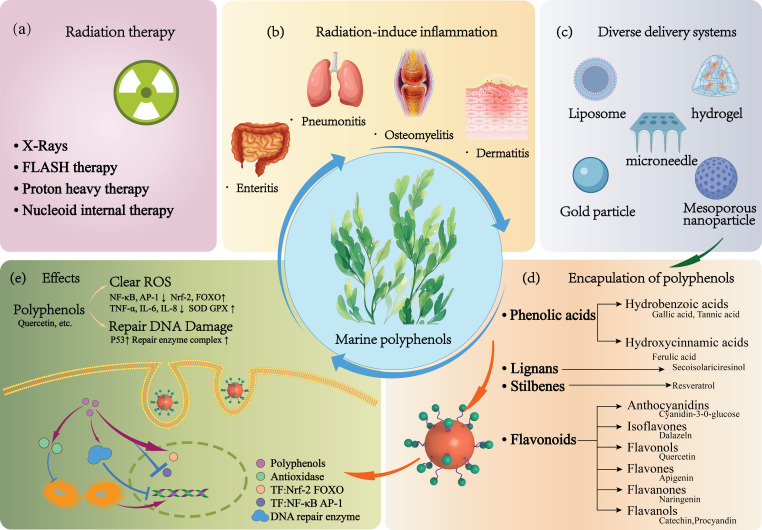
Schematic overview of polyphenol delivery systems for enhanced radiation protection and treatment efficacy. (**a**) Spectrum of prevalent radiation therapies; (**b**) principal categories of radio-induced inflammation; (**c**) varied delivery systems for marine polyphenols; (**d**) taxonomy of marine polyphenols based on chemical structure; (**e**) the mechanistic action of marine polyphenols in radioprotection.

The distinctive antioxidant and bioactive attributes of marine polyphenolic compounds present considerable potential for advancement in the functional food industry. Nonetheless, their molecular structural characteristics create prevalent challenges during food processing and digestion, such as poor water solubility and vulnerability to oxidative degradation, which restrict their bioactivity retention and functional expression [101]. Consequently, nano-delivery systems utilizing marine-derived carriers, capitalizing on their structural compatibility, represent a fundamental technological approach for improving the stability of marine polyphenols and achieving targeted functionality. Concurrently, the controlled release of bioactive components facilitated by these systems can synergistically enhance the effectiveness of regulating intestinal microenvironment homeostasis (Figure 3) [102].

At the molecular level, fucoxanthin-a marine carotenoid with polyphenolic properties-exhibits significant antioxidant activity due to its long-chain conjugated double bonds and epoxy group structure. Nonetheless, this configuration also makes it highly susceptible to light, temperature, and digestive enzymes [12]. A study creatively employed kelp-derived nanocellulose and sodium caseinate to formulate fucoxanthin nanoparticles, examining their efficacy in mitigating oxidative stress and curtailing lipid accumulation. Oxidation facilitated by 2,2,6,6-tetramethylpiperidine-1-oxyl (TEMPO) resulted in uniformly distributed kelp-derived nanocellulose. At a TEMPO-oxidized kelp nanofiber-to-sodium caseinate mass ratio of 1:3, in vitro experiments revealed that these nanoparticles demonstrated remarkable biocompatibility and were effectively absorbed by cells, significantly improving Fx bioavailability [103]. This enhanced delivery efficacy mitigates oxidative stress by activating Nrf2 via signaling pathways such as Akt/GSK-3β/Fyn or Nrf2/HO-1/NQO1, thereby effectively inhibiting excessive free fatty acid (FFA)-induced lipid droplet formation. Additionally, in vivo distribution studies in mice confirmed the effective accumulation of nanoparticles in the intestine and liver, demonstrating their potential for targeted drug delivery. The kelp nanocellulose-TKNC/SC (kelp nanocellulose/chitosan) composite system has been identified as an optimal protective carrier for marine polyphenols due to its biocompatibility and distinctive structural characteristics [104].

Nanocellulose, derived from the cell walls of brown algae, possesses a one-dimensional nanofiber architecture characterized by porous channels and a high specific surface area, facilitating the encapsulation of marine polyphenols via physical adsorption and hydrogen bonding. Hydroxyl groups on the fiber surface establish stable hydrogen bond networks with the phenolic hydroxyl groups of brown algae polyphenols. Additionally, the porous fiber structure provides protection against oxygen and light exposure, thereby significantly diminishing the oxidation rate of polyphenols [103]. In contrast, the storage capacity of 2,2,6,6-Tetramethylpiperidine-1-oxyl (TEMPO)-oxidized kelp nanocellulose (TKNC-Fx) was significantly higher than that of kelp nanocellulose. This notable enhancement in stability was primarily ascribed to the effective encapsulation of Fx by TKNC. In simulated gastric fluid, the degradation rate of free Fx increased rapidly, indicating that it was easily degraded in an acidic environment. However, the Fx degradation of TKNC-Fx was slow and stable due to the protection of cellulose, and the Fx release rate only increased after entering the simulated intestinal fluid. This slow-release effect helps the active ingredient to be released stably in the intestine, improves its bioavailability, and avoids premature decomposition in the stomach [103,105].

At the functional expression level, the precise release of marine polyphenols mediated by delivery systems synergistically enhances the efficacy of gut microbial environment homeostasis regulation [106]. After encapsulation in orally delivered microspheres, fucoxanthin achieves targeted release in specific intestinal segments. Its conjugated double bond structure improves the local microenvironment by regulating intestinal redox balance. Research confirms that when applied in functional food matrices, this system significantly elevates antioxidant factor levels within the gut [107]. This mechanism is intrinsically linked to fucoxanthin’s structural properties: its polyene conjugated system scavenges reactive oxygen species through electron transfer, while its epoxy group undergoes addition reactions with intestinal free radicals, thereby maintaining gut microenvironment homeostasis [108]. The biomimetic cell wall model TEMPO-oxidized cellulose nanofiber (TCNF) was used to enhance the environmental stability of liposomes and play a role in the acid response mechanism. The encapsulation technique can protect the structure of liposomes and Fx in the acidic environment of the stomach. The biomimetic cell wall is designed to disperse and release Fx in simulated intestinal fluid. The results showed that the release rate of 0.5%TCNF@Fx-liposome was only 7.9% after 2 h of digestion in simulated gastric fluid, and the release rate of Fx in Fx-liposome was 17.55% higher than that of 0.5%TCNF@Fx-liposome. In the simulated intestinal fluid, the release rate of Fx-LIP was maintained at about 25%, which indicated that Fx-liposome had stopped releasing Fx in the simulated intestinal fluid, while the release rate of 0.5%TCNF@Fx-liposome reached 67.61% after 1 h [109]. Notably, the core value of the aforementioned functions lies in maximizing the functional expression of marine polyphenols within food matrices through structural protection and targeted delivery [110].

### 3.3. Metabolic Regulation of Marine Lipids in the Gut Microbiome

Marine lipids exhibit exceptional nutritional properties due to their unsaturated bond configurations and distinctive functional groups. Nonetheless, their molecular structure results in challenges such as low absorption efficiency and vulnerability to metabolic degradation. Furthermore, the mechanisms that regulate their interactions with gut microbiota remain inadequately elucidated, constraining their use in functional foods aimed at regulating metabolic homeostasis [111]. On the mechanism of direct microbiota modulation, although the amphiphilic structure of astaxanthin has certain interfacial activity, its conjugated double bond chain is prone to isomerization during digestion. Furthermore, free astaxanthin is readily degraded by intestinal microbiota, leading to a bioavailability of less than 20% [112]. Marine lipids can regulate metabolic homeostasis by modulating gut microbiota composition and signaling pathways, with their mechanisms of action intricately linked to their molecular structural characteristics [113]. The polyunsaturated double bond structure of DHA influences gut microbiota composition by affecting membrane fluidity; studies indicate that incorporating microalgae-derived DHA into food matrices decreases the relative abundance of pathogenic bacteria (e.g., *E. coli*), while enhancing beneficial bacteria (e.g., *Bifidobacteria*, *Lactobacillus*). The primary mechanism involves DHA’s double bond configuration, which inhibits the membrane synthesis pathways of pathogenic bacteria while supplying appropriate lipid metabolic substrates for beneficial bacteria [114].

On the barrier effect mechanism, DHA can improve intestinal oxidative stress by regulating the expression of factors associated with the Nrf2/HO-1 signaling pathway. Its cis-double bond structure functions as a signaling molecule to activate Nrf2 nuclear translocation, thereby initiating HO-1 expression and achieving redox balance in the intestinal microenvironment. This effect has been validated by functional food research and is achieved entirely through nutritional activity [115]. The amphiphilic nature of astaxanthin allows it to concurrently affect gut microbiota and signaling pathways. Its polar terminal interacts with polysaccharide groups in the intestinal mucosa, creating a protective barrier that prevents the adhesion of pathogenic bacteria [116]. Concurrently, its conjugated double bond chain scavenges reactive oxygen species in the gut, synergistically modulating metabolic homeostasis via the Nrf2/HO-1 pathway [117].

**Figure 4:**
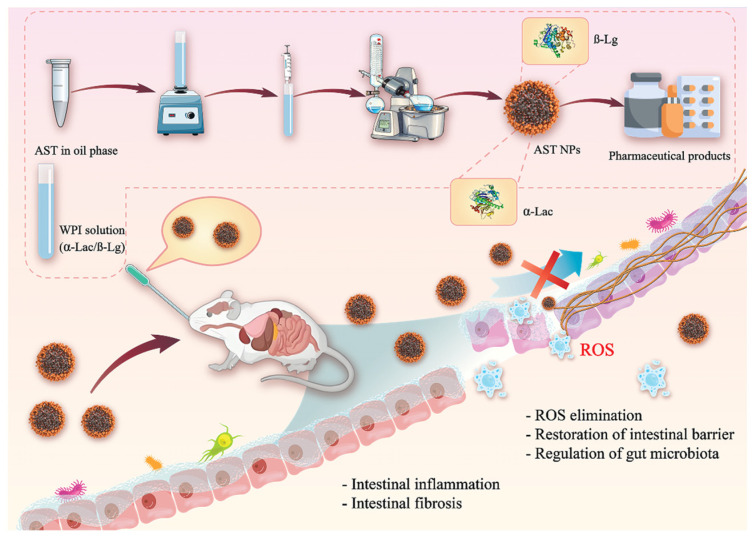
Schematic illustration of AST NPs preparation process and their anti-inflammatory/antifibrotic effects. AST NPs were prepared by the emulsion-solvent evaporation method. After oral administration, whey protein isolate protected AST from gastric juice, and AST was slowly released from NPs at lesion sites to alleviate intestinal inflammation and fibrosis via a synergistic therapeutic effect. ROS: Reactive Oxygen Species.

On the mechanism of delivery-mediated effects, recent advancements have been made in optimizing absorption strategies for marine lipids and elucidating their metabolic regulation mechanisms associated with microbiota. The primary strategy focuses on enhancing absorption efficiency through delivery systems while utilizing molecular structural characteristics to achieve targeted regulation of gut microbiota and signaling pathways [118]. The absorption disorder of marine lipids is intrinsically linked to their molecular structure. DHA, as a kind of ω-3 long-chain polyunsaturated fatty acid, is easily hydrolyzed by lipase in the intestine and lost due to its six cis-unsaturated double bonds. Its hydrophobic structure also hinders its passage through the intestinal mucosal barrier. In the in vitro absorption model, emulsified DHA showed the highest FFA absorption rate, which was significantly higher than free DHA and encapsulated DHA (10.7%, *p* < 0.05) [119]. The bioavailability of DHA is influenced by various factors, including its release characteristics, lipolysis, and the absorption of microcapsules within the gastrointestinal tract. These factors are contingent upon decisions made during the encapsulation process, such as the choice of carrier materials and the source of DHA. The wall materials typically employed to encapsulate DHA-rich oils are crucial in preventing DHA from degradation in the stomach and facilitating its diffusion in the intestine, thereby enhancing absorption [120]. Intestinal permeability is a key factor affecting the bioavailability and physiological efficacy of DHA in microcapsules. However, how DHA microcapsules are transformed and how the components are absorbed by the small intestinal membrane need to be further studied. Some studies have established an in vitro absorption model based on the permeability of the rat small intestine to evaluate the intestinal absorption of DHA microcapsules after in vitro digestion. The absorption rate of FFA released by microcapsules was the highest due to the rapid accumulation of substances on the intestinal wall. In addition, DHA microcapsules with algal oil as the source of DHA had a higher absorption ratio of FFA in the in vitro model than tuna oil. The composition of the carrier material seems to have little effect on the absorption of FFA [121]. Natural AST is commonly extracted from Haematococcus pluvialis, crustaceans, and bird feathers by corresponding appropriate methods. Solvent extraction, oil stripping, enzymatic hydrolysis, supercritical fluid extraction and microwave-assisted extraction are mostly applied to accumulate AST with high yields. Delivery systems such as emulsions, nanoparticles and liposomes may improve the stability and bioavailability of AST [122]. The oral liver-targeted nanoparticles optimize astaxanthin absorption by achieving targeted enrichment through the compatibility of surface-bound ligands with intestinal and hepatic tissues. Their core-shell structure protects the ketone and hydroxyl groups of astaxanthin from degradation. Concurrently, the biodegradable shell permits controlled release of the active ingredient, augmenting astaxanthin retention in the liver by 4.2 times relative to free forms [123]. Astaxanthin demonstrates remarkable antioxidant capabilities with a high safety profile; however, its utilization is limited by inadequate solubility and vulnerability to oxidation. Astaxanthin nanoparticles (NPs) were synthesized and systematically validated through molecular dynamics simulations and in vitro property characterization to identify a viable oral nanocarrier with potential application value [124]. The pH-sensitive and mucosal-adherent astaxanthin nanoparticles, formulated with whey protein isolate, securely protect astaxanthin from gastric degradation and facilitate targeted release at sites of intestinal inflammation (Figure 4) [125].

In conclusion, the advancement of marine polyphenol delivery systems and the metabolic regulation of marine lipids depend on their molecular structural attributes and compatibility with marine-derived carriers. These technologies offer theoretical foundations for the high-value utilization of marine bioactive compounds in functional foods, with their primary value based on the enhancement of nutritional and homeostasis-regulating efficacy of marine-derived ingredients via structural optimization and functional synergy [126].

## 4. Application of Marine Bioactive Substances in Gut Microbiota

### 4.1. Design of Intestinal Microbial Delivery Systems

The design objective of the intestinal microecological delivery system is to protect marine bioactive substances as they traverse the gastrointestinal barrier, ensuring their targeted release within the gut and efficient interaction with the microbiota [127]. Marine-derived bioactive substances possess both biological safety and functional synergy, serving as the fundamental components of the delivery system. The marine origin of these carriers drives the system’s design [128]. Sodium alginate is a natural polysaccharide derived from brown algae. Owing to its superior gelling characteristics and ion sensitivity, it is extensively utilized in the encapsulation of probiotics and bioactive substances [129]. Investigations indicate that when sodium alginate and chitin oligosaccharide are employed as wall materials to deliver *Escherichia coli* Nissle 1917 (EcN) via this double-layer microcapsule system. This mechanism stems from oligosaccharide chains enhancing capsule wall density through hydrogen bonds and electrostatic interactions while providing a carbon source for probiotics [130]. Fucoidan, another crucial marine polysaccharide, exhibits stable electronegativity across a pH range of 2.0–12.0. This property enables them to form composite carriers with sodium caseinate through electrostatic self-assembly. These carriers delay the release of active ingredients in simulated gastric conditions while rapidly disintegrating under intestinal pH conditions, achieving precise regulation [131]. Improving the delivery system for marine-derived bioactive compounds can enhance their functional value. Among these, the configuration characteristics directly determine the loading efficiency, release kinetics, and bioavailability of the active ingredients [108]. The design must be informed by the solubility of the compounds, functional targets, and properties of the food matrix. The common delivery systems in functional foods are microspheres and microcapsules [132]. These systems generally employ marine polysaccharides, such as sodium alginate and chitosan, as carriers, which are prepared through emulsification-crosslinking or spray drying techniques, with particle sizes ranging from 1 to 100 μm [133]. Their spherical hollow structure provides a physical barrier against high temperatures and acid-base stress during food processing, demonstrating excellent encapsulation compatibility for water-soluble marine fucoxanthin and other components [134]. By adjusting the crosslinking degree, these systems can achieve pH-responsive functionality, maintaining structural stability in the stomach while swelling and releasing drugs in the intestine. Nanoparticles and nanocapsules measuring between 10 and 50 nm possess distinct benefits for substances necessitating trans-membrane absorption, including omega-3 polyunsaturated fatty acids [135]. Marine phospholipid/fish oil-based nanocapsules, utilizing their amphiphilic structure, have advanced in applications for encapsulating lipophilic components in dairy products and oral liquids [136]. Liposomes and nanomulsions utilizing squid lecithin and fish oil phospholipids as membrane constituents exhibit dual-phase component loading capabilities [137]. The bilayer lipid membrane emulates biological membrane structures to improve the effectiveness of intestinal cell fusion, while the hydrophobic core establishes an antioxidant milieu that considerably prolongs the shelf life of readily oxidized components such as astaxanthin and DHA [138]. The electrospun nanofibers, with diameters ranging from 50 to 500 nm, derived from fish gelatin and sodium alginate, possess a high specific surface area and porosity, rendering them suitable for the co-loading of marine polysaccharides and probiotics. Their flexible architecture accommodates solid matrices such as gel candies without introducing undesirable taste sensations [139]. Concurrently, sodium alginate and κ-carrageenan-based gel microspheres establish three-dimensional networks via Ca^2+^ ion cross-linking. Their exceptional water retention and pH-responsive swelling characteristics render them optimal carriers for water-soluble components, such as marine oligosaccharides, in beverages and yogurt [140].

The progressive trajectory for delivery systems is rooted in functional synergistic design. Marine polysaccharide carriers not only provide protective functions, but their prebiotic properties can also create synergistic effects with encapsulated active substances [141]. Researchers have confirmed that sodium alginate-based microcapsules promote the proliferation of beneficial gut bacteria such as *lactobacilli* and *bifidobacteria*. This synergistic effect, combined with the microbiota’s enzyme-modulating action, enhances the fermentation of relatively short-chain fatty acid metabolites, thereby exerting anti-inflammatory effects in mouse models. This ternary interaction model involving the carrier, active ingredient, and microbiota provides a research foundation for designing delivery systems [142].

### 4.2. Applications of Interventions Related to Gut Microbiome Dysregulation

The characteristics of gut microbiota dysbiosis are manifested as reduced microbial diversity, decreased abundance of beneficial bacteria, and imbalanced metabolic products. Marine bioactive substances exert their effects by reshaping microbial communities, regulating metabolic pathways, and improving host phenotypes, exhibiting source specificity and diverse mechanisms of action [143].

On the mechanism of direct microbiota modulation, marine polyphenolic compounds exhibit potent microbiota-modulating activity. Compared to polyphenols from other sources, marine polyphenols exhibit higher stability in the gut and longer-lasting interactions with microbiota due to their rich resorcinol structure [144]. Marine lipids can regulate the gut microbiota microbiome through multidimensional mechanisms, providing critical support for the microbiome-modulating functions of functional foods [145]. It primarily consists of omega-3 polyunsaturated fatty acids (EPA, DHA) and marine phospholipids. At the microbial level, EPA and DHA selectively enrich beneficial bacteria such as *bifidobacteria* and *lactobacilli* while inhibiting the colonization and proliferation of opportunistic pathogens like *Enterobacteriaceae* and *Clostridium* species. This enhances microbial diversity and community stability [146].

On the barrier effect mechanism, marine polysaccharides modulate the gut microbiota through molecular structural intervention [48]. The intervention mechanism of *Sargassum fusiforme* fucoidan (SFF) has been systematically elucidated. The rising global obesity rate urgently demands new strategies. Research has elucidated the indirect mechanism underlying the metabolic benefits of SFF. The administration of SFF to obese mice resulted in a 6.3-fold increase in serum tauroursodeoxycholic acid, despite no direct effects on adipocytes in vitro. This elevation of TUDCA induced by SFF was identified as a crucial mediator in improving systemic insulin resistance (IR) and inflammation in white adipose tissue. Mechanistic investigations demonstrated that TUDCA directly mitigates palmitate-induced lipotoxicity in adipocytes by activating the G protein-coupled bile acid receptor 5 and its downstream cAMP-PKA signaling pathway. This activation inhibited NF-κB-driven inflammation and curtailed the catabolism of pro-inflammatory arachidonic acid/linoleic acid [147]. A study examines the mechanisms by analyzing alterations in gut microbiota composition, gene expression, and metabolites in mice subjected to a high-fat diet, following intervention with SF and SFF, utilizing 16S rRNA sequencing, transcriptomics, and non-targeted metabolomics. The results obtained demonstrate that *Sargassum fusiforme* mitigates obesity induced by a high-fat diet by reducing the abundance of specific *Firmicutes phylum* bacteria, decreasing levels of glycochenodeoxycholic acid, glycoursodeoxycholic acid, glycohyodeoxycholic acid, and glycodeoxycholic acid, and modulating the expression of the Elovl3, Gm3734, Per2, and Tnnc1 genes. This offers a novel perspective on treatment strategies for hypercholesterolemia and introduces new nutritional intervention approaches to lower cholesterol levels [148]. Marine phospholipids promote bile acid metabolism and conversion within the gut microbiota, generating secondary bile acids to strengthen the intestinal barrier and enhance the synthesis of beneficial metabolites such as short-chain fatty acids. This process improves the host’s gut microbiome balance, providing crucial theoretical support for the development of marine-derived functional foods [149].

On the mechanism of delivery-mediated effects, certain studies have utilized the modified exosome of Phaeodactylum tricornutum (*P. tricornutum*) and other constituents to establish an Fx-based targeted delivery system. The objective is to enhance the bioavailability, stability, and targeted transport of Fx. The findings indicated a marked improvement in the uptake and targeting efficiency of RAW264.7 macrophages. There was a reduction in oxidative cellular damage and an induction of macrophage polarization compared to free Fx. The activity assessment of the Fx delivery system confirmed the structural integrity, in vitro storage properties, gastrointestinal stability, and regulatory effect on lipopolysaccharide induced inflammatory cells [150]. Resistant starch serves as a dietary fiber impervious to enzymatic digestion in the small intestine, allowing it to reach the colon where it is fermented by colonic microbiota, resulting in the production of short-chain fatty acids. Concurrently, micelles formed by the interaction of phospholipids and resistant starch can solubilize and transport Fx, thereby enhancing metabolic and colonic health benefits. Microparticles of resistant starch can be synthesized by recrystallizing short-chain glucan through the enzymatic differentiation of starch protein, exhibiting significant resistance to digestive enzymes within the human gastrointestinal tract. Research indicates that the encapsulation efficiency of delivering Fx via resistant starch microparticles and supplementary components can reach approximately 91%. Furthermore, resistant starch microparticles facilitate the controlled release of Fx in various intestinal regions, including the duodenum, jejunum, ileum, and colon. This approach offers novel concepts for innovative delivery systems and demonstrates promising application potential [151].

The evaluation system for intervention effects has become increasingly diversified. Currently, the research is no longer confined to analyzing microbial community structure but integrates cutting-edge technologies to achieve a comprehensive analysis from structural characterization to functional assessment [152]. The structure-activity relationship models of bioactive compounds constructed using artificial intelligence technology enable predictive evaluation of regulatory effects across diverse intestinal microenvironments, facilitating optimal parameter selection through assessment outcomes [153]. The utilization of 3D-printed biomimetic microcarriers enhances the assessment framework as a standard for metrics like targeted delivery efficiency and sustained-release kinetics [154]. The combined application of metagenomics, metabolomics, and other multi-omics methodologies can clarify alterations in microbial metabolites (e.g., short-chain fatty acids) following active ingredient intervention and their interaction mechanisms with the intestinal barrier, offering comprehensive scientific evidence for the intervention effects of marine-derived functional foods [155].

### 4.3. Precision Delivery System Technological Innovation

The structural attributes of marine-derived bioactive compounds confer intrinsic structure-activity benefits for functional delivery technologies, facilitating the accurate targeting of bioactive substances via delivery systems [156]. The essential technology of marine-derived active ingredient delivery systems is rooted in the targeted development and meticulous design of intelligent responsive delivery carriers, which need to achieve structural compatibility and functional synergy with marine-derived bioactive compounds [157]. Additionally, the controlled release mechanisms of these active ingredients permit the regulation of targeted release within designated microenvironments [158].

Researchers designed a dual-responsive system utilizing metal-phenolic nanoparticles as core carriers. By facilitating coordination interactions between epigallocatechin gallate and Fe^3+^, Fx was encapsulated, resulting in a pH/ROS dual-responsive mechanism that enables targeted Fx release at inflammatory sites. The surface modification with chondroitin sulfate-cystamine-triphenylphosphonium bromide provides dual targeting capabilities to macrophages and mitochondria. This is achieved through the specific affinity of chondroitin sulfate for the CD44 receptor, which is highly expressed on activated macrophages, in conjunction with the mitochondrial targeting attributes of triphenylphosphonium, thereby enhancing Fx accumulation within diseased cells [99]. Research investigations have shown that a nano-system utilizing alginate carriers and hyaluronic acid to deliver anthocyanins facilitates the visualization of inflammation-targeted areas. Alginate effectively prevents gastric acid leakage, significantly blocks reactive oxygen species damage to RAW264.7 cells, and prevents mitochondrial membrane potential decline. This system precisely targets the CD44 receptor on the surface of colonic inflammatory cells and achieves highly efficient accumulation at sites of intestinal inflammation in vivo [159].

### 4.4. Regulatory Challenges and Conversion Bottlenecks

The innovation in precision delivery technology centers on molecular recognition mechanisms. By leveraging the interaction between specific functional groups in marine bioactive substances and gut microbiota or epithelial cells, precise targeting is achieved [160]. Research indicates that coupling sulfated galactose residues from fucoidan with lectins on probiotic surfaces creates a conjugation system. This study provides an innovative strategy for developing precision delivery systems and highlights the potential of orally administered hydrophobic bioactive substances in inflammatory interventions [161]. The conversion of marine bioactive compounds into precisely formulated gut nutrition products encounters several fundamental challenges. At the regulatory level, international standards lack consistency; while the U.S. FDA classifies certain algal polysaccharides as GRAS substances, the EFSA has not established specific daily intake limits for novel bioactive elements like fucoidan. The management of marine biotoxins requires adherence to HACCP systems; however, there is a deficiency of a specialized regulatory framework for bioactive substances, leading to heightened product compliance expenses [58].

The scalability of food-grade applications is constrained by the availability of raw materials and processing capabilities. While aquatic byproducts provide material support, environmentally sustainable and standardized extraction methods for bioactive compounds remain scarce. Large-scale production often leads to the degradation of bioactivity, and the substantial costs associated with processing hinder industrial transformation [162]. Regarding safety, in addition to conventional risks such as heavy metals and solvent residues, concerns about the potential toxicity of components like low-molecular-weight carrageenan highlight risks that are contingent upon structure and dosage. Research on the long-term effects of consumption on gut microbiota homeostasis is insufficient, and the regulation of beneficial bacterial abundance lacks clinical validation [163].

Significant gaps exist between laboratory settings and real food matrices. Static in vitro digestion models fail to accurately mimic the dynamic biochemical milieu and mechanical shear forces present in the human body. Although dynamic models more closely resemble physiological conditions, they are complex and expensive to operate. Furthermore, numerous studies overlook the influence of food matrix components, such as proteins and lipids, on the release and absorption of bioactive compounds, leading to considerable divergences between in vitro results and in vivo realities. Tackling these challenges requires interdisciplinary collaboration to promote gut-targeted nutritional applications of marine bioactive compounds [164].

Functional foods, serving as carriers for active ingredients, require optimization through technologies such as nanocarriers and pH-responsive delivery systems to overcome the bottlenecks of low bioavailability and weak targeting. Only then can they precisely match individual metabolic differences and achieve personalized nutritional interventions. However, current human clinical data on the combined application of functional foods with precision nutrition and delivery systems remains significantly insufficient, creating a bottleneck for industrialization in this field [21]. Therefore, supplementing high-quality human clinical data is a core future research direction. Efforts should focus on expanding sample coverage, extending intervention periods, establishing multidimensional evaluation systems, and clarifying the relationship between delivery system optimization and individual efficacy.

## 5. Conclusions

Marine bioactive compounds possess high application value due to their unique functional properties. Their structural and functional diversity demonstrates irreplaceable potential in the field of microecological regulation, yet large-scale development and precise control still face multiple practical challenges. In actual application, the large-scale acquisition of marine-derived bioactive compounds is constrained by the sustainability of marine biological resources and the efficiency of separation and purification technologies. Furthermore, the complex interference of factors makes it difficult to maintain their active steady state. In microbiome regulation, the interaction mechanisms and regulatory pathways between marine-derived bioactive compounds and microbial communities require further elucidation. The directional enrichment effects on dominant functional microbial communities, inhibition thresholds for harmful microorganisms, and dose-response relationships demand a standardized quantitative assessment. The future progress of microecological regulation could concentrate on bioactive substances produced from marine sources, utilizing technological innovation to tackle problems. Artificial intelligence approaches can be utilized to develop data models, improving the correlation between compound structures and regulatory functions, while forecasting regulatory effectiveness under various environmental situations to enhance research efficiency. Three-dimensional printing technology facilitates the creation of bionic microcarriers for precise distribution and prolonged release of marine-derived bioactive compounds, hence improving their enduring effectiveness inside microecosystems. The omics method of design can integrate several omics platforms, including metagenomics and metabolomics, to elucidate the interaction processes between marine-derived bioactive chemicals and microbial metabolic pathways, thus offering molecular evidence for precision-targeted design. The meticulous design of delivery systems utilizing functional synergy strategies can enhance the development paradigm of marine-derived bioactive compounds from conventional resource exploration to multi-target precision regulation, thereby facilitating the industrialization of functional foods for microecological regulation.

## Figures and Tables

**Table 2:** The main categories of marine polyphenols and lipids.

Polyphenol Types	Applications	Ref.
Brown algae polyphenols	PT-protein-chitosan microencapsulation of tomato seed oil for food and other related fields	[72]
Fucoxanthin	Construction of oral dual-responsive targeted microspheres for the intervention of ulcerative colitis	[99]
**Lipid types**	**Applications**	**Ref.**
DHA	Emulsion gel co-delivery of Fe^2+^ and DHA for fortified foods	[100]
Astaxanthin	Oral liver-targeted nanoparticles for astaxanthin delivery to alleviate alcoholic liver injury	[93]

## Data Availability

No new data were created or analyzed in this study. Data sharing is not applicable to this article.

## Supplementary Materials

The following supporting information can be downloaded at: https://www.mdpi.com/article/10.3390/foods15030545/s1, Table S1. Summary of key values related to marine bioactive substances.
