# Supplementary material for: Marine Bioactive Substances in Precision Nutrient Delivery to the Gut and Advances in Microbiome Regulation: A Narrative Review
Source: Foods. 2026 Feb 4;15(3):545. doi: 10.3390/foods15030545 (PMC12896926; doi:10.3390/foods15030545)
Supplement: Supplementary file 1 [file foods-15-00545-s001.zip › foods-4114179-supplementary.pdf]

Supporting Information

Marine Bioactive Substances in Precision Nutrient Delivery to the Gut and Advances in Microbiome Regulation: A Narrative Review

Xue Zhao [1,2], Shan Huang [1,3], YaWei [1,2], DiWang [1,2], Chunsheng Li [1,2], Chuang Pan [1,2], Yueqi Wang [1,2], Huan Xiang [1,2], Gang Yu [1,2,]* and Yongqiang Zhao [1,2,]*

[1] Key Laboratory of Aquatic Processing, Ministry of Agriculture and Rural Affairs, National Research and Development Center for Aquatic Product Processing, South China Sea Fisheries Research Institute, Chinese Academy of Fishery Sciences, Guangzhou 510300, China; zhaoxue@scsfri.ac.cn (X.Z.); 21240713121@stu.ouc.edu.cn (S.H.); weiya@scsfri.ac.cn (Y.W.); wangdi@scsfri.ac.cn (D.W.); lichunsheng@scsfri.ac.cn (C.L.); panchuang@scsfri.ac.cn (C.P.); wangyueqi@scsfri.ac.cn (Y.W.); xianghuan@scsfri.ac.cn (H.X.)

[2] Key Laboratory of Efficient Utilization and Processing of Marine Fishery Resources of Hainan Province, Sanya Tropical Fisheries Research Institute, Sanya 572426, China

[3] College of Food Science and Engineering, Ocean University of China, Qingdao 266000, China

* Correspondence: gyu0928@163.com (G.Y.); zhaoyq@scsfri.ac.cn (Y.Z.); Tel.: +86-20-89108346 (Y.Z.)

Table S1. Summary of key values related to marine bioactive substances.

Category	Specific Indicator	Value	Related Substance/System	Ref.
Marine Polysaccharides	Probiotic Survival Rate (28 days storage at -20°C)	≥71.3%	Chitosan Hydrogel Delivery System	[61]
	Probiotic Survival Rate (28 days storage at 4°C)	≥55.1%	Chitosan Hydrogel Delivery System	[61]
	Fucoidan Branching Degree Threshold	≥30%	Fucoidan from Brown Algae (recognized by Bacteroides species)	[62]
	Lutein Encapsulation Efficiency	87.3%–94.2%	Phenolic Acid-Grafted Agarose	[63]
	Viable Probiotics in Simulated Gastric Fluid	＞10^6 CFU	Sodium Alginate-Chitooligosaccharide Double-Layer Microcapsules (delivering Escherichia coli Nissle 1917)	[130]
	Viable Probiotics in 4% Bile Acid	10^9 CFU	Sodium Alginate-Chitooligosaccharide Double-Layer Microcapsules (delivering Escherichia coli Nissle 1917)	[130]

Marine Polyphenols	Tomato Seed Oil Microencapsulation Efficiency	Up to 91.2%	Phlorotannin-Pea Protein Isolate-Chitosan Ternary Composite Wall Material	[72]
	Intracellular Fucoxanthin Content	18.7 mg/g Dry Weight	Haematococcus pluvialis (Cultivated in Artificial Photobioreactor)	[74]
	Fucoxanthin Encapsulation Efficiency	84.81%	N-Acetylgalactosamine-Modified Probiotic Vesicle Delivery System	[76]
	In Vitro Bioavailability of Fucoxanthin	65.77%	N-Acetylgalactosamine-Modified Probiotic Vesicle Delivery System	[76]
	Fucoxanthin Encapsulation Efficiency	82.2%	Kelp Nanocellulose-Sodium Caseinate Complex (Mass Ratio 1:3)	[103]
	Fucoxanthin Storage Retention Rate (14 days)	56.12%	Kelp Nanocellulose-Sodium Caseinate Complex	[103]
	Retention Rate After 5 h UV Irradiation	56.33%±2.52%	TEMPO-Oxidized Kelp Nanocellulose-Fucoxanthin Complex	[105]
	Retention Rate After 150 min Heating	57.33%±0.58%	TEMPO-Oxidized Kelp Nanocellulose-Fucoxanthin Complex	[105]
	Release Rate in Simulated Gastric Fluid (2 h)	7.9%	0.5%TCNF@FX-Liposomes	[109]
	Release Rate in Simulated Intestinal Fluid (1 h)	67.61%	0.5%TCNF@FX-Liposomes	[109]
	Encapsulation Efficiency	Approximately 91%	Resistant Starch Microparticle-Fucoxanthin Delivery System	[151]
Marine Lipids	DHA Purity	35%–45%	Microalgae-Derived (Schizochytrium sp., Crypthecodinium cohnii Fermentation)	[87]
	DHA Purity	15%–20%	Deep-Sea Fish Extract	[87]
	DHA Release Rate	91.7%±1.3%	Small-Volume Liposomes (delivering Lactoferrin and DHA)	[88]
	Free DHA Release Rate	64.6%±3.4%	Unencapsulated DHA	[88]
	Astaxanthin Production Efficiency	Up to 45 mg/g Dry Weight	Haematococcus pluvialis (Cultivated in Closed Photobioreactor)	[92]
	Astaxanthin Targeted Retention Rate	76.2%	Oral Liver-Targeted Nanoparticles	[93]
	Intestinal FFA Absorption Rate (Emulsified DHA)	16.2%	Emulsified DHA (In Vitro Absorption Model)	[119]

	Intestinal FFA Absorption Rate (Free DHA)	11.2%	Free DHA (In Vitro Absorption Model)	[119]
	Intestinal FFA Absorption Rate (Microencapsulated DHA)	26.6%	DHA Microcapsules (In Vitro Absorption Model)	[121]
	FFA Absorption Rate of Algal Oil-Derived DHA Microcapsules	36.40%	Algal Oil-Derived DHA Microcapsules (In Vitro Model)	[121]
	FFA Absorption Rate of Tuna Oil-Derived DHA Microcapsules	14.26%	Tuna Oil-Derived DHA Microcapsules (In Vitro Model)	[121]
	Astaxanthin Bioavailability	$<$20%	Free Astaxanthin (Prone to Degradation by Intestinal Microbiota)	[112]
	Probiotic Survival Rate (21 days storage at 4°C)	7 log CFU/g	Fucoidan-Polyvinyl Alcohol Electrospun Nanofibers (loading Lactiplantibacillus plantarum)	[161]
